# Diterpenoid compounds from *Wedelia trilobata* induce resistance to *Tomato spotted wilt virus* via the JA signal pathway in tobacco plants

**DOI:** 10.1038/s41598-019-39247-6

**Published:** 2019-02-26

**Authors:** Lihua Zhao, Zhonghui Hu, Shunlin Li, Xueping Zhou, Jing Li, Xiaoxia Su, Lizhen Zhang, Zhongkai Zhang, Jiahong Dong

**Affiliations:** 1 Institute of Biotechnology and Germplasm Resources, Yunnan Academy of Agricultural Sciences, Yunnan Provincial Key Laboratory of Agricultural Biotechnology, Key Lab of Southwestern Crop Gene Resource and Germplasm Innovation, Ministry of Agriculture, 650204 Kunming, China; 2 Kunming Institute of Botany, Chinese Academy of Science, 650201 Kunming, China; 3 Institute of Biotechnology, Zhejiang University, Hangzhou, 310058 Zhejiang China; 4 Life Science College, Southwest Forestry University, 650224 Kunming, China

## Abstract

*Tomato spotted wilt virus* (TSWV) causes major losses of many crops worldwide. Several strategies have been attempted to control disease caused by TSWV. However, many challenges for the effective control of this disease remain. A promising approach is the use of abiotic or biotic inducers to enhance plant resistance to pathogens. We screened a diterpenoid compound from *Wedelia trilobata*, 3α-Angeloyloxy-9β-hydroxy-*ent*-kaur-16-en-19-oic acid (AHK), which had higher curative and protective effects against TSWV than the ningnanmycin control. The rapid initiation of the expression of all the TSWV genes was delayed by more than 1d in the curative assay, and the expression of the *NSs*, *NSm* and *RdRp* genes was inhibited. In addition, the replication of all TSWV genes in systemic leaves was inhibited in the protective assay, with an inhibition rate of more than 90%. The concentrations of jasmonic acid (JA) and jasmonic acid isoleucine (JA-ILE) in the AHK-treated and systemic leaves of the treated plants were significantly higher than those observed in the control. The results suggested that AHK can induce systemic resistance in treated plants. The transcription of the *NtCOI1* gene, a key gene in the JA pathway, was significantly higher in both the inoculated and systemic leaves of the AHK-treated plants compared to the control. The AHK-induced resistance to TSWV in *Nicotiana benthamiana* could be eliminated by VIGS-mediated silencing of the *NtCOI1* gene. These results indicated that AHK can activate the JA pathway and induce systemic resistance to TSWV infection.

## Introduction

*Tomato spotted wilt virus* (TSWV) belongs to the genus *Orthotospovirus* and is transmitted by thrips (Thysanoptera) in a propagative manner^1^. TSWV is a destructive plant virus with a wide host range, infecting more than 1000 plants species from 90 families and is considered to be one of the top ten economically important plant viruses worldwide^2,3^. Despite developing many TSWV control measures, including insecticide application, the use of resistant cultivars, phytosanitation, and cultural control tactics, successful control is not readily achieved in many agricultural systems owing to the absence of resistant varieties or resistance breaking and the resistance of thrips to insecticides^4^.

The use of abiotic or biotic inducers is an effective way to enhance plant resistance to pathogens. Several natural or chemical products have been described as potential elicitors of induced resistance to diseases^5,6^. However, the antiviral efficacies of these compounds against systemic infections of plant viruses are unknown, with the exceptions of acibenzolar-S-methyl (BTH), eugenol, 3-acetonyl-3-hydroxyoxindole (AHO), and chitosan oligosaccharide (COS)^7–10^. These compounds have been shown to effectively enhance tobacco (*Nicotiana xanthi* NN, *N*. *tabacum*) and tomato resistance to systemic infection by TSWV and *Tobacco mosaic virus* (TMV), respectively, through induction of systemic acquired resistance (SAR). BTH is a signaling molecule that can enhance the expression of defense genes and elicits an increase in the concentration of H_2_O_2_ in treated leaves^7^. Defense activation by BTH, AHO, and COS in response to viral infection is supported by an induction of the salicylic acid (SA) signaling pathway, which is the primary biochemical marker of the systemic acquired resistance (SAR) transduction pathway^7–10^. Eugenol enhances the resistance of tomato plants to *Tomato yellow leaf curl virus*(TYLCV) by stimulating the production of endogenous nitric oxide (NO) and SA and by regulating the expression of *SlPer1*, a host *R* gene specific to TYLCV in tomato plants^11,12^. Phytohormones, such asjasmonic acid (JA), SA, and abscisic acid (ABA), can enhance resistance to pathogens during plant defense responses^13–15^.

*Wedelia trilobata* (L.) Hitchc. [syn. *Sphagneticola trilobata* (L.) Pruski] (creeping oxeye) is native to the tropics of Central America. Recently, this notoriously invasive weed has invaded India, South China, and Japan^16^. The results of some studies have suggested that compounds extracted from *W*. *trilobata* have pharmacological functions in medical treatments and biological activities in agriculture^17,18^, including anti-TMV^19^, insect toxin^20^, fungistatic, and bacterial inhibitory activities^19,21^. However, little is known regarding compounds from *W*. *trilobata* with anti-TSWV inhibition mechanisms. In this study, 28 diterpenoid compounds extracted from *W*. *trilobata* were screened using the lesion counting method. The results showed that 3α-Angeloyloxy-9β-hydroxy-*ent*-kaur-16-en-19-oic acid (AHK) had the highest activity against TSWV by eliciting the JA signaling pathway and elicited induced systemic resistance (ISR) in tobacco.This approach may be an effective and environmentally friendly way to control TSWV infections.

## Results

### Compound structural analysis

3*α*-Angeloyloxy-9*β*-hydroxy-*ent*-kaur-16-en-19-oic acid (10.4 mg), a white powder, was shown to have the molecular formula C_25_H_36_O_5_ as determined by the results of ESI-MS, ^13^C NMR and DEPT analyses. Comparison of the 1D NMR spectroscopic data of 1 with those of 3*α*-Angeloyloxy-9*β*-hydroxy-*ent*-kaur-16-en-19-oic acid revealed that they shared an identical structure that was determined to be 3*α*-angeloyloxy-9*β*- hydroxy-*ent*-kaur-16-en-19-oic acid^22^.

3*α*-Angeloyloxy-9*β*-hydroxy-*ent*-kaur-16-en-19-oic acid: white powder, C_25_H_36_O_5_; ESI-MS m/z: 439 [M + Na]^+^, ^1^H NMR (CDCl_3_, 400 MHz) *δ* (ppm): 6.01 (1 H, dd, *J* = 13.8, 6.8 Hz, H-3′), 4.75 (1 H, s, H-17a), 4.71 (1 H, s, H-17b), 4.58 (1 H, dd, *J* = 11.9, 4.1 Hz, H-3), 2.64 (1 H, d, *J* = 17.5 Hz, H-15a), 2.57 (1 H, s, H-13), 1.24 (3 H, s, H-18), 1.11 (3 H, s, H-20); ^13^C NMR (CDCl_3_, 100 MHz) *δ* (ppm): 180.6 (s, C-19), 167.6 (s, C-1′), 154.7 (s, C-16), 138.1 (d, C-3′), 127.9 (s, C-2′), 103.4 (t, C-17), 78.4 (d, C-3), 77.3 (s, C-9), 49.2 (d, C-5), 48.9 (s, C-8), 47.9 (s, C-4), 43.68 (s, C-10), 43.65 (t, C-15), 42.1 (d, C-13), 40.3 (t, C-14), 35.8 (t, C-7), 34.4 (t, C-12), 30.6 (t, C-1), 30.0 (t, C-11), 24.1 (q, C-18), 23.9 (t, C-2), 21.4 (t, C-6), 20.7 (q, C-5′), 17.2 (q, C-20), 15.7 (q, C-4′).

### Curative effect assay

**Figure 1:**
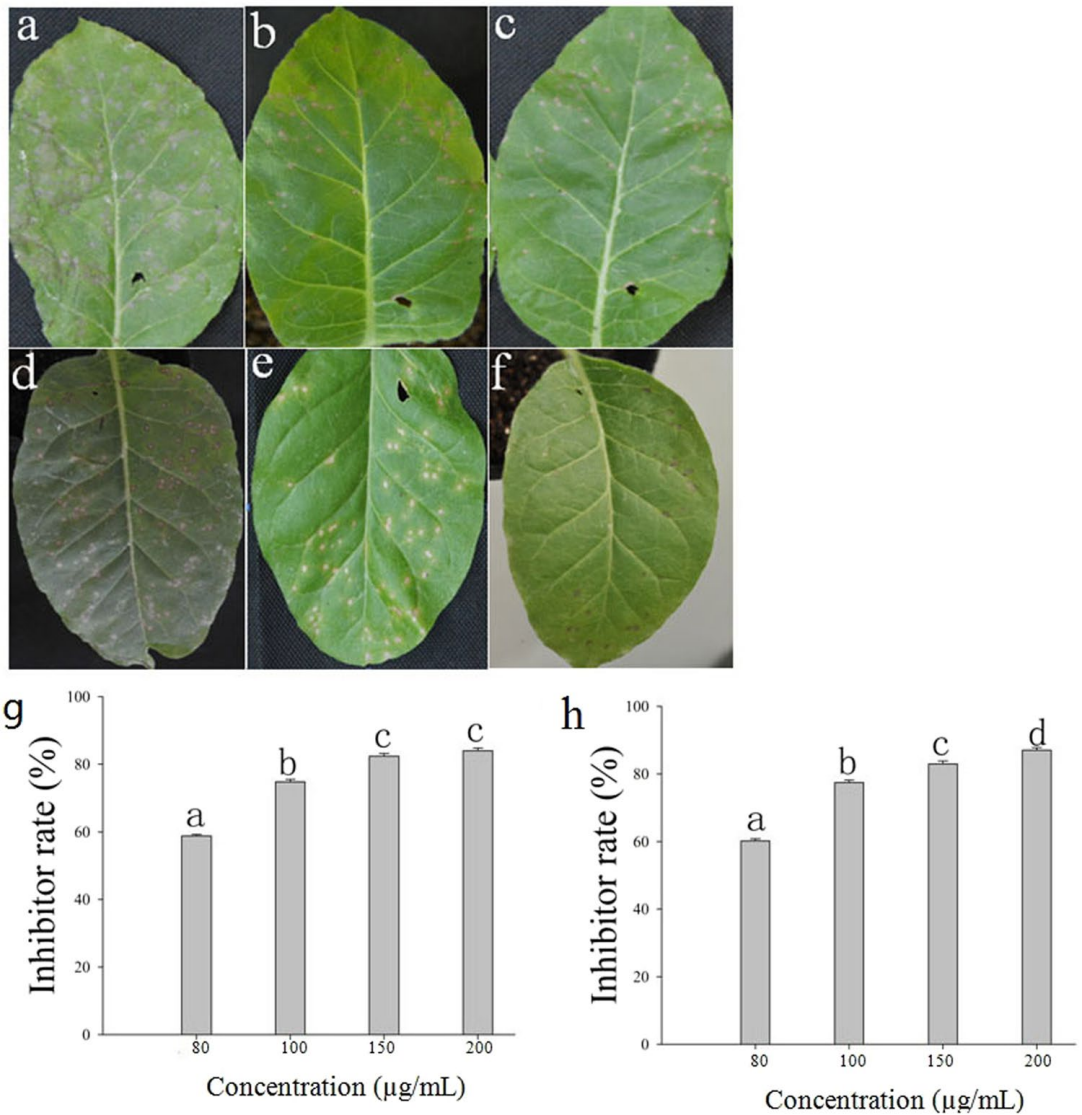
Inhibitory effect of anti-TSWV with AHK different treatments. (**a**–**c**) The curative effect of AHK. Image (**a**) shows the positive control (treatment with TSWV and H_2_O), image (**b**) shows the ningnanmycin control (treatment with TSWV and ningnanmycin), and image (**c**) shows the AHK treatment (treatment with TSWV and AHK); (**d**–**f**) The protective effect of AHK. Image (**d**) shows the positive control (treatment with H_2_O and TSWV), image (**e**) shows the ningnanmycin control (treatment with ningnanmycin and TSWV), and image (**f**) shows the AHK treatment (treatment with AHK and TSWV); Image (**g**) shows the curative effect of AHK at different concentrations; Image (**h**) shows the protective effect of AHK at different concentrations. Different letters indicate significant differences among the compounds compared with ningnanmycin; differences are considered significant at P ≤ 0.05.

The activities of 28 diterpenoid compounds from *W*. *trilobata* against TSWV were tested by counting lesions on inoculated leaves at 3 days post inoculation (dpi). An examination of the inhibition rates revealed that 15 of these compounds had varying degrees of curative effects, whereas the other 13 compounds had no inhibitory effect. Compounds 8, 9, 13, 16, 20, 25, 27 and 28 had higher curative inhibition rates than that of the control ningnanmycin (52.48%) at a concentration of 100 μg/mL. Compound **20**, 3α-Angeloyloxy-9β-hydroxy-*ent*-kaur-16-en-19-oic acid (AHK), had the highest inhibition rate (62.40%) at a concentration of 100 μg/mL (Fig. 1a–c and Table 1).

**Table 1:** The inhibition rates of TSWV with treatment by different compounds.

Compound name	Inhibitor rate (%)		Compound name	Inhibitor rate (%)	
	Protection	Curation		Protection	Curation
Compound1	56.50 ± 1.90b	54.80 ± 0.70b	Compound16	66.30 ± 0.70b	61.60 ± 0.60b
Compound2	53.30 ± 0.90b	45.80 ± 0.30b	Compound18	57.20 ± 2.80b	47.80 ± 3.10b
Compound3	58.10 ± 0.40b	46.30 ± 0.20b	Compound20	76.50 ± 0.40b	62.40 ± 1.70b
Compound6	58.80 ± 1.10a	56.00 ± 0.70a	Compound22	60.50 ± 0.40a	52.10 ± 1.20b
Compound7	54.80 ± 0.70b	52.40 ± 1.60b	Compound25	63.20 ± 1.60a	61.30 ± 2.10b
Compound8	54.40 ± 0.70b	60.80 ± 1.30a	Compound27	67.10 ± 0.20b	62.20 ± 0.20b
Compound9	50.00 ± 0.40b	61.90 ± 1.60b	Compound28	69.50 ± 0.60b	61.90 ± 0.30b
Compound13	58.4 ± 0.90a	60.30 ± 1.10b	Ningnanmycin	61.25 ± 0.70a	58.80 ± 1.30a

Different letters indicate significant differences for different compounds compared with ningnanmycin; differences are significant at P ≤ 0.05.

### Protective effect assay

The results of this assay revealed that 15 of the 28 diterpenoid compounds assayed had varying degrees of activity against TSWV, whereas the other 13 compounds had no inhibitory effect. Compounds 20, 22, 25, 27 and 28 had higher protective inhibition rates than that of the control ningnanmycin (61.25%) at a concentration of 100 μg/mL. AHK had the highest inhibition rate (76.50%) (Fig. 1d–f and Table 1).

### Concentration gradient assay of AHK

The results of a necrotic lesion inhibition assay demonstrated that the activity of AHK against TSWV in tobacco K326 increased with increasing concentrations of AHK (80, 100, 150 and 200 µg/mL). At a concentration of 200 μg/mL, AHK was observed to exhibit the highest *in vivo* curative and protective effects (84.0 and 87.0%, respectively) among the concentrations tested (Fig. 1g,h).

### AHK inhibition of gene expression of TSWV *in vivo*

**Figure 2:**
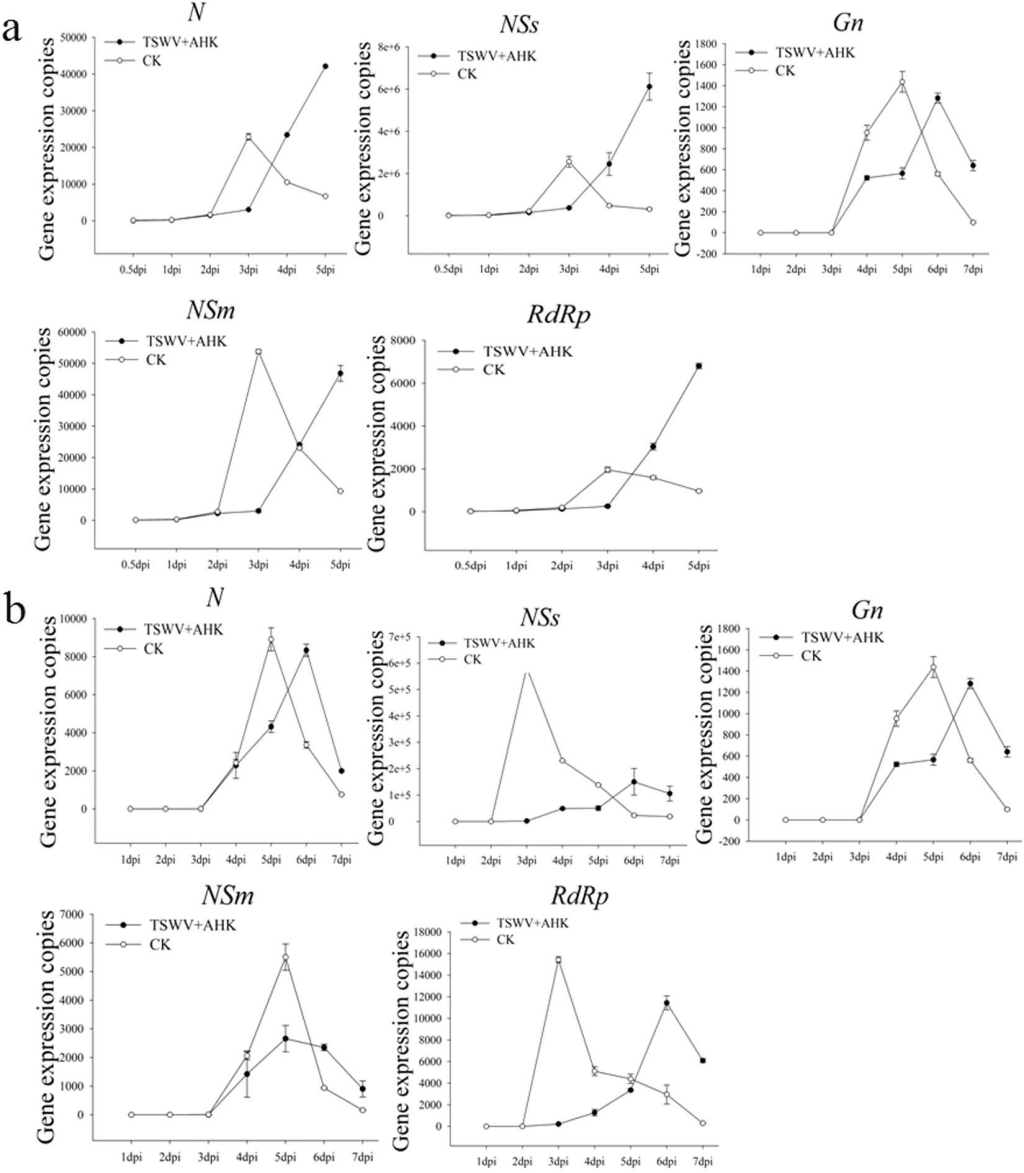
Levels of TSWV gene expression in AHK-treated leaves to assess the curative effect of AHK. Image (**a**) presents the level of TSWV gene expression with and without the AHK treatment in inoculated K326 leaves in the curative effect assay using qRT-PCR; Image (**b**) presents the level of TSWV gene expression with and without the AHK treatment in K326 systemic leaves in the curative effect assay using qRT-PCR; CK: positive control (TSWV-infected tobacco).

The transcription levels of the TSWV genes *Gn*, *N*, *NSs*, *NSm* and *RdRp* were detected by qRT-PCR in the AHK-treated and systemic upper leaves for the *in vivo* curative assay. Sampling time was determined by the development of symptoms, and sampling ceased when the degree of leaf necrosis was very serious or when the leaf withered. The results showed that the rapid initiation of expression of all TSWV genes was delayed by 1 day (postponed from 2 to 3 dpi) in the inoculated leaves of the AHK- treated plants. Compared with untreated control leaves, the peak expression of the *N*, *G*n, *NSs*, and *RdRp* genes in the treatment leaves was delayed from 3 to 5 dpi (Fig. 2a). The degree of leaf necrosis was used to determine the viral titer, and as leaf withering increased, the replication of the viral genes also decreased (Table S4).

In contrast to the *N*, *Gn*, *NSm*, and *RdRp* genes, the expression of the *NSs* gene was lowest in the systemic leaves. The inhibition rate of *NSs* gene expression was as great as 74.15% and was higher than other genes (*N*, 6.40%; *Gn*, 10.79%; *NSm*, 57.33%; and *RdRp*, 25.88%). The rapid replication initiation for *NSs* and *RdRp* genes was delayed by 3 days from 2 to 5 dpi (Fig. 2b and Table S3). The results may demonstrate that systemic infection by TSWV was inhibited in the AHK-treated plants by inhibition of the expression of *NSs*.

**Figure 3:**
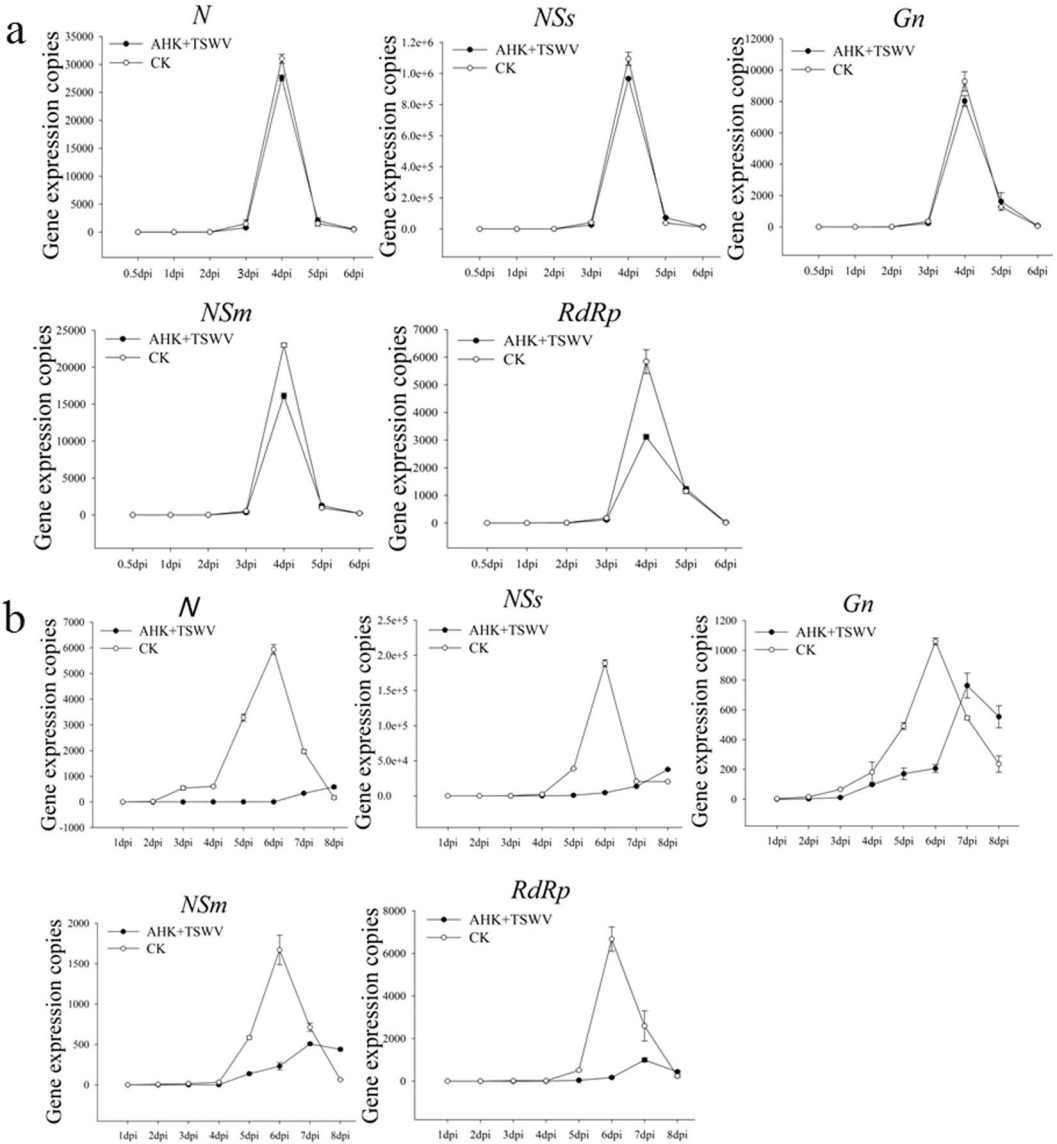
Levels of TSWV gene expression in AHK-treated leaves to assess the protective effect of AHK. Image (**a**) presents the level of TSWV gene expression with and without the AHK treatment in inoculated K326 leaves assayed by qRT-PCR; Image (**b**) presents the level of TSWV gene expression with and without the AHK treatment in systemic K326 leaves in the protective effect assay using qRT-PCR; CK: positive control (TSWV-infected tobacco).

In addition, the transcription levels of TSWV genes *Gn*, *N*, *NSs*, *NSm*, and *RdRp* were detected by qRT-PCR in the AHK-treated leaves and in the systemic upper leaves for the protective assay *in vivo*. In the inoculated leaves, the results showed that the expression of all TSWV genes in the treatment and control plants had the same trend of a rapid initiation of expression at 2 dpi, peaking at 3 dpi and followed by an abrupt decrease. AHK treatment had no any effect on the expression of TSWV genes except for *RdRp* in the protective assay (Fig. 3a).

In the systemic upper leaves of the control plants, all TSWV genes exhibited rapid expression initiation at 4 dpi, peaking at 6 dpi and decreasing thereafter. However, the expression of all TSWV genes in the AHK-treated plants remained at a low level until 8 dpi, when the leaves of control plants exhibited a very serious degree of necrosis or were withered, and had inhibition rates from 69.61 to 90.14%, except for *Gn* (27.91%) (Fig. 3b and Table S3). These results demonstrated that systemic movement of the virus was inhibited in the protective assay.

### Effects of AHK on N and NSm protein expression

**Figure 4:**
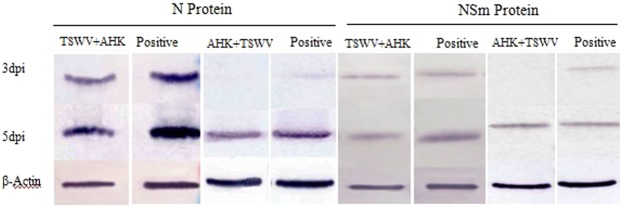
Expression of the TSWV N and NSm proteins. The expression of the N and NSm proteins with the AHK treatment in the protective and curative effect assays on the inoculated leaves by western blot; positive control (TSWV-infected leaves).

The expression of both N (a structural protein) and NSm (a nonstructural protein) was detected by western blot at 0.5, 1.5, 3, and 5 days post inoculation(dpi) in the inoculated leaves from the curative and protective assays (Fig. 4). The results showed that the expression of the N and NSm proteins were not detected at 0.5 dpi, 1.5 dpi, and the expression of the N protein in the curative and protective assays was lower than that observed in the positive control (TSWV-treated leaves) at 3 and 5 dpi, while the expression of the N protein at 5 dpi was higher than at 3 dpi. The expression of the NSm protein in the curative assay was lower than that of the positive control at 3 and 5 dpi, while the expression of the NSm protein in the protective effect assay was lower than that of the positive control on 3 dpi, but no difference was observed on 5 dpi. Taken together, these results show that the expression of the N protein was inhibited after AHK treatment in both the curative and protective assays, while the expression of the NSm protein was notably inhibited in the curative assay but not in the protective assay.

### Effects of AHK on phytohormone concentration in host leaves

**Figure 5:**
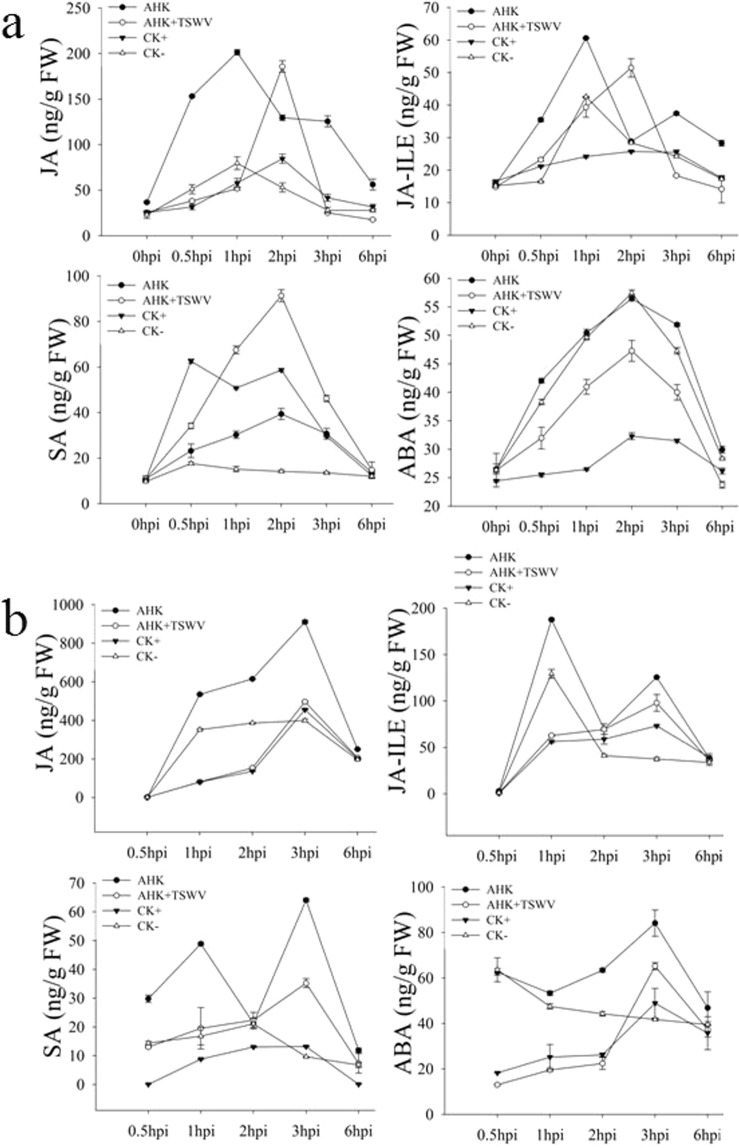
Concentrations of hormones after AHK treatment. Image (**a**) shows the concentrations of phytohormones (JA, JA-ILE, SA, and ABA) in inoculated leaves at 0, 0.5, 1, 2, 3, and 6 hpi; image (**b**) shows the concentrations of phytohormones (JA, JA-ILE, SA, and ABA) in systemic upper leaves at 0.5, 1, 2, 3, and 6 hpi; AHK: treatment with AHK; AHK + TSWV: inoculation of TSWV at 6 h post-AHK treatment; CK+: positive (TSWV inoculated); CK-: negative (DMSO treatment).

Plant phytohormones are closely associated with plant resistance to viral infections. The concentrations of JA, jasmonic acid isoleucine (JA-ILE), SA, and ABA in the inoculated leaves were determined to evaluate the defense-associated signaling pathway induced by AHK (Fig. 5a). The levels of both JA and JA-ILE in the AHK-treated inoculated leaves were generally higher than in the control leaves. The peak concentrations of JA and JA-ILE (AHK: 201.36 and 60.58 ng/g; AHK-TSWV: 185.80 and 51.47 ng/g, respectively) were 3 times higher than those observed in the control leaves. The concentration of SA in leaves from the AHK and AHK + TSWV treatments (AHK was smeared 6 h, after which TSWV was inoculated into the same leaves) peaked at 2 hpi and was higher than that observed in the control plant leaves (treated by DMSO), while the SA concentration in the AHK treatment leaves was lower than that observed in the leaves inoculated with TSWV. In contrast, the concentration of ABA in the treatment leaves did not increase compared with that observed in the control leaves. These results showed that AHK can significantly induce the production of JA and JA-ILE in the treated leaves. To confirm that the signals induced by AHK in the treated leaves were transferred into the systemic upper leaves, the concentrations of JA, JA-ILE, SA, and ABA in the systemic upper leaves were determined (Fig. 5b). The results showed that concentrations of JA and SA peaked at 3 hpi (910.49 and 64.08 ng/g, respectively) and were higher than those observed in the control plants (treated with TSWV or DMSO), and the concentration of JA was 14 times greater than that of SA. The concentration of ABA in the AHK-treated leaves was similar to that observed in the control leaves. Although the trends in the concentrations of JA, JA-ILE, SA, and ABA using the AHK + TSWV treatment were similar to the TSWV treatment, the concentrations of JA, JA-ILE, SA, and ABA in the AHK + TSWV-treated leaves were higher than in the TSWV treatment at 3 hpi, the same time that the JA concentrations peaked. These results indicated that the signals induced by AHK could be transferred into the systemic upper leaves, and for leaves pretreated with AHK, the pretreatment promoted a primed state of the plants^23^. Thus, when infected by TSWV, the plants could initiate the phytohormone-associated defense pathway to inhibit TSWV.

### Effects of AHK on the expression of key genes of different phytohormone signaling pathways

**Figure 6:**
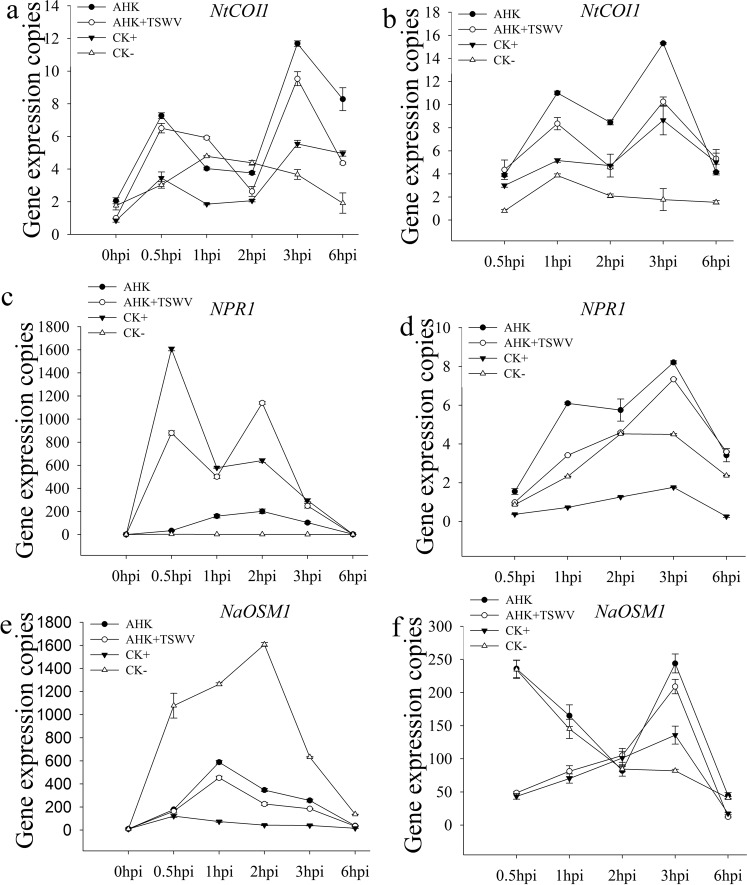
Transcription levels of key genes of the JA, SA, and ABA signaling pathways. (**a**) Shows the transcription level of the *NtCOI1* gene of the JA pathway in inoculated leaves; (**b**) shows the transcription level of the *NtCOI1* gene of the JA pathway in systemic upper leaves; (**c**) shows the transcription level of the *NPR1* gene of the SA pathway in inoculated leaves; (**d**) shows the transcription level of the *NPR1* gene of the SA pathway in systemic upper leaves; (**e**) shows the transcription level of the *NaOSM1* gene of the ABA pathway in inoculated leaves; (**f**) shows the transcription level of the *NaOSM1* gene of the ABA pathway in systemic upper leaves. AHK: AHK treatment; AHK + TSWV: inoculation of TSWV at 6 h post-AHK treatment. CK+: positive (TSWV inoculated); CK-: negative (DMSO treatment).

To further confirm the key signal pathways involved in the AHK-induced resistance to TSWV, we examined the transcription of the JA signal pathway marker gene *NtCOI1*, which is the key component of ubiquitin-ligase complexes (SCF^COI1^)^24–26^, the transcription of the *NPR1* gene of the SA signal pathway, and the transcription of the *NaOSM1* gene of the ABA signal pathway. The qRT-PCR analysis showed that the AHK and AHK + TSWV treatments induced higher expression of the *NtCOI1* gene in the inoculated leaves and in the systemic upper leaves than was observed in the control treatment. These results indicated that signal induced in the inoculated leaves was transferred to the upper leaves and was amplified. The AHK treatment had no effect on the transcription of the *NPR1* and *NaOSM1* genes. The TSWV infection caused the SA levels to increase at 0.5 hpi, although the signal was not transferred from the treated leaves to the upper leaves (Fig. 6). These results showed that the JA signaling pathway was activated by AHK and played a key role in the resistance to TSWV infection.

### Loss of AHK-induced resistance to TSWV after silencing the *NtCOI*1 gene

**Figure 7:**
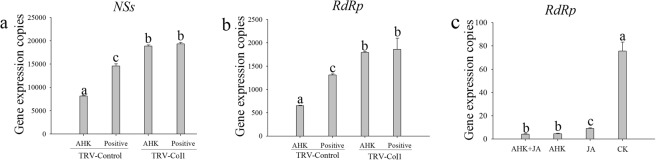
Effective anti-virus activity of AHK after silencing the *COI1* gene. (**a**) Shows the replication level of the *NSs* gene in the curative effect assay in the TRV-Control and TRV-CoI1 tobacco; (**b**) shows the replication level of the *RdRp* gene in the protective effect assay in the TRV-Control and TRV-CoI1 tobacco; (**c**) shows the transcription level of the *RdRp* gene in the silenced tobacco and inoculated with TSWV 6 h after different treatments; AHK: AHK treatment; positive: samples only inoculated with TSWV; AHK + JA: silenced tobacco and treatment with AHK and JA; JA: silenced tobacco and treatment with JA; CK: Silenced tobacco, inoculated with TSWV JA. Differences are considered significant at P ≤ 0.05.

The transcription levels of the *NSs* gene in the curative assay and the *RdRp* gene in the protective assay were analyzed by qRT-PCR.The transcription levels of the *NSs* and *RdRp* genes were both significantly higher in the TRV-COI1 tobacco plants than in the TRV-Control tobacco plants (Fig. 7a,b). However, *RdRp* expression in the TRV-COI1 plants was significantly inhibited when the plants were sprayed with JA (Fig. 7c). Thus, the results indicated that AHK-induced resistance to TSWV depended on the JA signaling pathway.

## Discussion

Plant resistance to viruses can be induced by chemicals, such as benzothiadiazole, eugenol, 3-acetonyl-3-hydroxyoxindole (AHO), andsesquiterpenoids^10,12,27,28^. Induced resistance has been shown to be an alternative for managing viral infections of crops^29^. TSWV disease is one of the largest constraints on agricultural production because of the lack of pesticides against TSWV and the extensive adaptability of its vector. In this study, we used the tobacco-TSWV system to demonstrate that diterpenoid compounds from *W*. *trilobata* can induce systemic resistance to TSWV. The results of a lesion inhibition screening assay showed that of the 28 diterpenoid compounds isolated from *W*. *trilobata*, 100 µg/mL of AHK had a higher inhibitory activity than the control ningnanmycin, which has been widely used in agriculture to control TMV and other viral diseases^30,31^. In assays testing the curative and protective effects of AHK, the inhibition rates obtained using 100 and 200 µg/mL of AHK were 62.40 and 61.25% and 84.00 and 87.00%, respectively.

To complete a successful infection cycle in the host plant cell, a virus must enter into the cell of the host plant and undergo uncoating, translation and replication of the genome followed by encapsidation and multiplication before infecting neighboring cells and spreading systemically. Replication of the viral genome is one of the key steps in the completion of a successful infection cycle. We performed qRT-PCR to detect the replication level of five genes encoded by TSWV. Compared with the controls, the rapid initiation of the expression of all the assayed genes was delayed by 1 day in the inoculated leaves of the AHK-treated plants. In the systemic upper leaves, the expression of the *NSs*, *NSm* and *RdRp* genes was maintained at a lower level in the AHK-treated plants compared to the control, with gene expression inhibition rates of greater than 90% observed together with a delay in rapid expression initiation by 3 days. The NSs protein, a viral suppressor of RNA silencing, plays a key role in the replication and transcription of viral genes and is necessary for the establishment and maintenance of systemic infection in plants^32,33^. NSm, the movement protein, is also related to symptom development^34–36^, while RdRp is a replicase of viral RNAs^37,38^. These results indicated that AHK-treated plants could activate RNA silencing and block the movement and replication of viruses to defend against TSWV infection. RNA silencing is regarded as pattern-triggered immunity (PTI) against viruses. AHK had no effect on the replication level of the membrane glycoprotein (*Gn/Gc*) gene of TSWV. The results also demonstrated that the Gn/Gc protein, which is involved in viral transmission by thrips, did not play a key role in TSWV infection of tobacco plants. The observed inhibition rates of the replication of TSWV genes demonstrated that AHK had more of protective effect than curative effect.

The plant hormones SA, JA, ABA, and ethylene (ET) play key roles in regulating signaling networks associated with plant defense toward pathogens. SA is required for SAR activation in tissues distal from the site of infection, while jasmonate and ethylene are indispensable for ISR. SAR and ISR, two primary forms of plant defense, can be activated when plants are infected by pathogens, e.g., bacteria, fungi, and viruses, or by abiotic agents, e.g., elicitors, which may result in resistance against subsequent infections by pathogens^13,39^. For example, in TMV-infected tobacco, the transcript level of genes associated with the JA-mediated defense pathway was significantly upregulated after 1 day, whereas genes associated with the SA-mediated defense pathway were significantly upregulated after 5 days^13^. Some chemicals, including the cyclodipeptides cyclo (L-Pro-L-Pro) and cyclo (D-Pro-D-Pro), chitosan, schisanhenol derivatives, and a glycoprotein of BDP-30 inhibited TMV infection through activation of SA- or JA-induced systemic resistance^9,40–43^. In our study, after AHK treatment of tobacco leaves, the accumulation of JA and JA-ILE significantly increased in both the treated leaves and in the systemic upper leaves and was higher than that observed for SA and ABA. The level of SA accumulation in the TSWV-inoculated leaves was higher than that observed in the uninoculated leaves, and the SA signal was not transduced into the systemic upper leaves (Figs 5a,b-SA and 6c,d). The results of the qRT-PCR analysis showed that the transcription of *NtCOI1*, which plays a key role in the JA pathway, was higher in both inoculated and systemic leaves than was observed in the controls. AHK-induced resistance to TSWV could be eliminated by VIGS-mediated silencing of *NtCOI1*. Thus, the results indicated that AHK-induced systemic resistance to TSWV was dependent on the JA signaling pathway, not the SA signaling pathway. Although TSWV infection could induce the SA signaling pathway, the SA signal could be enhanced under AHK induction (Fig. 5a-SA). Additionally, TSWV is transmitted by thrips (order Thysanoptera), and the JA pathwayis required for resistance, predominantly against herbivorous insects^44^. Thus, the results of our study indicate that AHK, a diterpenoid compound from *W*. *trilobata*, is a strong candidate for the development of an environment-friendly pesticide for preventing TSWV diseases.

## Conclusions

In summary, this is the first report describing the use of 3α-Angeloyloxy-9β-hydroxy-*ent*-kaur-16-en-19-oic acid (AHK), a diterpenoid compound isolated and screened from extracts of *Wedelia trilobata*, to inhibit TSWV infection. In AHK-treated tobacco leaves, the rapid initiation TSWV gene expression was delayed by at least 1 day, the expression of the *NSs*, *NSm* and *RdRp* genes was inhibited, and the concentrations of jasmonic acid (JA) and jasmonic acid isoleucine (JA-ILE) increased significantly. The level of *NtCOI1* transcription in the JA signaling pathway increased in the AHK-treated plants. After *NtCOI1* gene silencing, AHK-induced systemic resistance to TSWV was lost. Our results indicated that AHK activates the JA pathway and induces systemic resistance to inhibit the expression of TSWV genes to inhibit infection of the plant by TSWV.

## Materials and Methods

### Plant material preparation

An entire *Wedelia trilobata* plant was collected in Xishuangbanna, Yunnan Province, China, on August, 2012. The specimen was identified by Yu Chen of Kunming Institute of Botany (KIB), Chinese Academy of Sciences (CAS). A voucher specimen (H20121005) has been deposited in the State Key Laboratory of Phytochemistry and Plant Resources in West China, Kunming Institute of Botany.

### Materials

The TSWV YN-Chili isolate was maintained and cultured in *Nicotiana tabacum* cv.K326. *N*. *tabacum* cv. K326 and *N*. *benthamiana* seeds were provided by theYunnan Academy of Tobacco Agricultural Science. Ningnanmycin was purchased from Heilongjiang DeQiang Biology Co., Ltd. The molecular structures of the pTV00 vector and the compounds are shown in Table S1.

### Compound separation and preparation

Dried powder of the whole *Wedelia trilobata* plant (22 kg) was extracted with MeOH (three times under reflux for 4, 4 and 3 h). Subsequently, the solvent was removed under reduced pressure to give a residue (2500 g, 11.4%), which was suspended with water and then successively extracted with petroleum ether, chloroform, and EtOAc. The extracts were evaporated under vacuum to produce the corresponding extracts of petroleum ether (903 g), chloroform (179 g), and EtOAc (70 g).

The petroleum ether extract was separated into 13 fractions(A-M) by silica gel column chromatography (100–200 mesh, 15 × 120 cm, 3000 g) and eluted with petroleum ether/EtOAc (v/v = 10:0, 9:1, 8:2, 7:3, 6:4, 5:5, and 0:10, 40 L each).

Fraction G (48 g) was separated on a silica gel G column(200–300 mesh, 10 × 100 cm, 1.65 kg) and eluted with petroleum ether/acetone (v/v = 19:1, 9:1,8:2, 7:3, and 6:4, 20 L each) to produce five fractions (1–5). Fraction 2 (7.18 g) was subjected to RP-18 silica gel column chromatography (20–45 µm, 30 × 460 mm, 150 g) and eluted with MeOH-H_2_O (v/v = 3:7, 5:5, 7:3, 8:2, and 9:1, 10 L each) and Sephadex LH-20 (CHCl_3_- MeOH, 1:1, 1.8 × 120 cm) to produce the compound AHK (20.4 mg) and other compounds.

### Curative assay *in vivo*

Twenty-eight diterpenoid compounds were extracted from *W*. *trilobata* and tested using the lesion counting method. The curative effect treatments were initiated with the inoculation of TSWV (100 μg/mL) onto whole *N*. *tabacum* cv. K326 leaves using cotton swabs. After 24 h, the diterpenoid compound solutions were spread onto the same area of the leaf that had been previously inoculated with TSWV. Two positive controls were used and involved the inoculation of tobacco leaves with TSWV (100 μg/mL) followed by the application of a ningnanmycin solution (80 µg/mL) or DMSO to the same leaf after 24 h, while leaves treated with DMSO alone were used as a negative control. The number of local lesions was recorded 3–4 days after inoculation, and three replicates were conducted for each sample. The compounds were dissolved in DMSO at a final concentration of 10 mg/mL and were diluted to 80, 100, 150 and 200 µg/mL with ddH_2_O.

The TSWV inhibition rates of the compounds were calculated according to the formula: inhibition rate (%) = [(T−C)/T] × 100%, where T is the average number of local lesions for the positive control and C is the average number of local lesions for the treatment.

### Protective assay *in vivo*

Twenty-eight diterpenoid compounds were tested using the lesion counting method. To examine protective effects of the compounds, solutions of the compounds were applied to whole *N*. *tabacum* cv. K326 leaves using cotton swabs. After 6 h, TSWV (100 μg/mL) was inoculated onto one leaf that had a compound applied previously. Two positive controls were included and involved the application of a ningnanmycin solution or DMSO to a leaf followed by inoculation with TSWV (100 μg/mL) onto the same leaf after 6 h, while leaves treated with DMSO alone were used as a negative control. The number of local lesions was recorded 3–4 days after inoculation, and three replicates were conducted for each sample.

### Quantitative RT-PCR

qRT-PCR was conducted to determine the number of copies of transcribed TSWV genes. Total RNA was extracted from tobacco leaves (0.2 g, fresh weight) using TriPure Isolation Reagent (Roche Diagnostics GmbH, Mannheim, Germany) according to the manufacturer’s instructions. Five replicates were performed for each sample. First-strand cDNA was synthesized using a One-Step gDNA Removal kit (Transgen, Beijing, China) followed by qRT-PCR using FastStart Universal SYBR Green Master (Rox) (Trans, Beijing, China) on an Applied Biosystems Stepone Plus instrument (Applied Biosystems, Foster City, CA, USA). The primer sequences used in this study are listed in Table S2.

A standard curve was established using plasmid DNA containing the corresponding gene sequence. The plasmid DNA was diluted for the standard samples with a dilution series (10^−2^, 10^−3^, 10^−4^, 10^−5^ and 10^−6^).

### Western blotting

The levels of the TSWV proteins N and NSm were analyzed by western blotting. Total protein was extracted from tobacco leaves (0.2 g, fresh weight) using TriPure Isolation Reagent (Roche Diagnostics GmbH, Mannheim, Germany) according to the manufacturer’s instructions. Equal sample volumes (20 μL) were loaded on a 12.5% polyacrylamide gel, and proteins were separated by electrophoresis at 100 V for 90 min. After being transferred to a PVDF membrane, the N and NSm proteins were detected using a primary antibody (1: 4,000) and were subsequently probed with AP-coupled goat anti-rabbit IgG (1:8,000; Sigma, Santa clara, USA). The signals on the membrane were visualized using a ready-for-use 5-bromo-4-chloro-3-indolylphosphate/nitroblue tetrazolium (BCIP/NBT) solution (Sangon Biotech, Shanghai, China).

### Phytohormone measurements

Phytohormones were extracted and quantified by LC-MS/MS as described previously^45^. Samples (approximately 150 mg) were ground to a fine powder in liquid nitrogen and briefly crushed, then 1 mL of ethyl acetate spiked with labeled internal standards (100 ng each of ^13^C_2_-JA, ^13^C_6_-JA-Ile, D_4_-SA, and D_6_-ABA) was added to each sample. After centrifugation at 13 000 g for 10 min at 4 °C, the supernatants were transferred to fresh tubes and evaporated to dryness using a vacuum concentrator (Concentrator plus, Eppendorf, Germany). Each residue was resuspended in 0.5 mL 70% (v/v) methanol and centrifuged (15 min, 13 000 g, 4 °C) to remove particles. The supernatants were analyzed on an HPLC-tandem mass spectrometry (1200 L LC-MS system, Varian, American). Five replicate leaf samples were used for each treatment.

### VIGS assays

A 217-bp fragment of the *COI1* cDNA sequence was amplified and cloned into pTV00^46^, and *Agrobacterium tumefaciens* (strain GV3101) carrying this construct was injected into *N*. *benthamiana*, generating *COI1*-silenced plants (VIGS COI1). To monitor the progress of VIGS, *phytoene desaturase* (*PDS*) was silenced, which eventually results in the visible bleaching of green tissues^45,47^ three weeks after injection. When the leaves of the *PDS*-silenced plants began to bleach and bolt, the youngest leaves of the VIGS COI1 and empty vector-inoculated plants were selected for curative and protective experiments. The samples used for qRT-PCR were collected after an additional 3 d. Five biological replicates per genotype were used for the experiments.

## Supplementary information

Dataset 1
