# Supplementary material for: Diterpenoid compounds from Wedelia trilobata induce resistance to Tomato spotted wilt virus via the JA signal pathway in tobacco plants
Source: Sci Rep. 2019 Feb 26;9:2763. doi: 10.1038/s41598-019-39247-6 (PMC6391457; doi:10.1038/s41598-019-39247-6)
Supplement: Supplementary file 1 — Dataset 1 [file 41598_2019_39247_MOESM1_ESM.pdf]

Supplementary Information

# Diterpenoid compounds from *Wedelia trilobata* induced resistance to *Tomato spotted wilt virus* via the JA signal pathway on tobacco plants

Lihua Zhao[1], Zhonghui Hu[2], Shunlin Li[2], Xueping Zhou[1, 3], Jing Li[4], Xiaoxia Su[1], Lizhen Zhang[1], Zhongkai Zhang[1], *, Jiahong Dong[1, *]

[1] Institute of Biotechnology and Germplasm Resources, Yunnan Academy of Agricultural Sciences, Yunnan Provincial Key Laboratory of Agricultural Biotechnology, Key Lab of Southwestern Crop Gene Resource and Germplasm Innovation, Ministry of Agriculture, Kunming 650204, China

[2] Kunming Institute of Botany, Chinese Academy of Science, Kunming 650201, China

[3] Institute of Biotechnology, Zhejiang University, Hangzhou, Zhejiang 310058, China

[4] Life Science College, Southwest Forestry University, Kunming 650224, China

* Correspondence: Jiahong Dong; Zhongkai Zhang

Phone/Fax: +86-870-6512-3133

Email: dongjhn@126.com (J. D.); zhongkai99@sina.com (Z. Z.)

Supplementary Table 1. The struction of compounds

| Compound number | Compound name | Compound struction |
|---|---|---|
| 1 | ManglisinA | 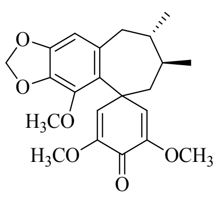 |
| 2 | 32-(Tiglinoyloxy)-162-17-dihydroxy-ent-kauran-19-oil-acid | 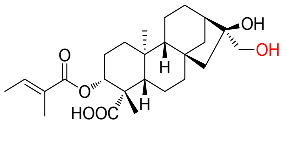 |
| 3 | Manglisin B | 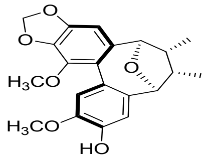 |
| 6 | Manglisin C | 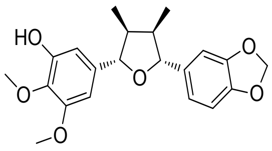 |
| 7 | (-)-4,7-aromadendranediol | 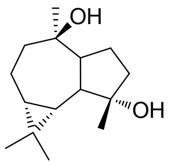 |
| 8 | Manglisin F | 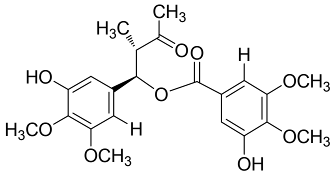 |
| 9 | Indol-3-carboxylic acid | 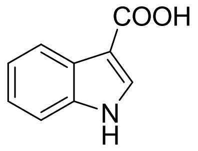 |

| | | |
|---|---|---|
| 13 | Manglisin G | 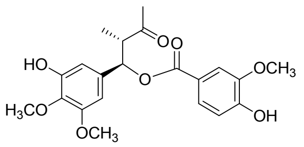 |
| 16 | Schinlignins B | 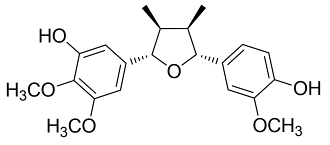 |
| 18 | Protocatecualdehyde | 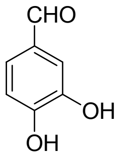 |
| 20 | 32-(Angeloyloxy)-9β-hydroxy-ent-kaur-16-en-19-oil acid | 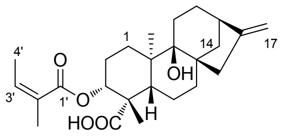 |
| 22 | Clemaphenol A | 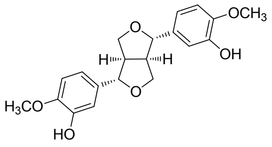 |
| 25 | Syringylglycerol | 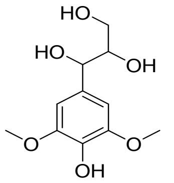 |
| 27 | Methy caffeoatte | 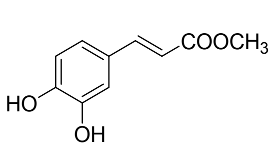 |
| 28 | 16α-17-dihydroxy-*ent*-kauran-19-oic acid | 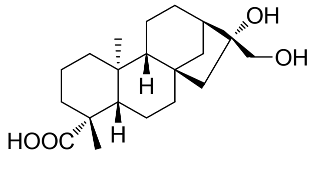 |

## Supplementary Table 2. Primers of qRT-PCR and VIGS

| Primer | Squence（5'-3'） | Base pai（bp） | Notes |
|---|---|---|---|
| TSWV-N-F | GTGAAGAAAGGGAAAGAGTATGCTGC | 26 | qRT-PCR |
| TSWV-N-R | GGGGTTAAAAAACAGGCAAAACTCAC | 26 | qRT-PCR |
| TSWV-NSs-F | TGCTCAAATACAGTCAATGCTAACG | 24 | qRT-PCR |
| TSWV-NSs-R | GGAGCTGGAATCGGTCTGTAATAT | 24 | qRT-PCR |
| TSWV-NSm-F | ATCTTGATGACCTTATTAGCGTT | 22 | qRT-PCR |
| TSWV-NSm -R | GAATGATAGTAGATACAAACCACCA | 24 | qRT-PCR |
| TSWV-RdRP-F | CCTTCAACTACAGGTATCCACTTCT | 23 | qRT-PCR |
| TSWV- RdRP -R | GGATTCTGCATACCCTTGTGA | 20 | qRT-PCR |
| TSWV-Gn-F | CTGTGGAAGTTCTGGTCAATAATG | 23 | qRT-PCR |
| TSWV- Gn -R | ATATAATCCCAGAAGCCCCAT | 19 | qRT-PCR |
| QCOI1-F | TTGTGCCAATTGGGCTTGAC | 20 | qRT-PCR |
| QCOI1-R | AGTTGGGACACCTTTGCAGT | 20 | qRT-PCR |
| VIGCOI1-F | CCC**AAGCTT**TTGGGGAATCCGATGAAGGC | 29 | Bold letters Hind III |
| VIGCOI1-R | CG**GGATCC**GGCAAGTATATGGGCGGGAT | 28 | Bold letters BamHI |
| M13F | CAGGAAACAGCTATGAC | 17 | |
| M13R | ACTGGCCGTCGTTTTAC | 17 | |
| pTV00F | CTAGTTCATCTGCACCGCCT | 20 | pTV00 |

Supplementary Table 3. Inhibitor rate of genes

| Gene names | Treatment | | | |
|---|---|---|---|---|
| | Curative effect | | Protective effect | |
| | Inoculated leaves | Systemic leaves | Inoculated leaves | Systemic leaves |
| *N* | No effect | 6.40% | 11.11% | 90.14% |
| *NSs* | No effect | 74.15% | 11.59% | 80.12% |
| *Gn* | No effect | 10.79% | 13.41% | 27.91% |
| *NSm* | No effect | 57.33% | 29.88% | 69.61% |
| *RdRp* | No effect | 25.88% | 46.64% | 85.05% |
